# A review of the DTS: Diathermic Syncope® system with a discussion regarding its use for kosher slaughter (*shechita*)

**DOI:** 10.1017/awf.2023.86

**Published:** 2023-10-02

**Authors:** Ari Z Zivotofsky

**Affiliations:** The Gonda Multidisciplinary Brain Research Center, Bar-Ilan University, Ramat Gan, Israel

**Keywords:** animal welfare, electrical stunning, fainting, humane slaughter, Jewish religious law, pre-slaughter stunning

## Abstract

Over the last several decades an alternative to current methods of stunning cattle has been developed. This system, DTS: Diathermic Syncope®, has been suggested to the Jewish and Muslim communities as a means to achieve pre-cut stunning in conformity with both religious and EU regulations without a need to resort to a derogation that permits an exemption from the EU requirement to pre-stun all animals undergoing slaughter. The developer’s contention is that the system induces fainting, and thus should be acceptable to all groups, including the kosher (Jewish) and Halal (Muslim) consumer. A review of the system based on publications and reports from the developer itself suggests that in reality the system selectively heats the brain, leading to an epileptic-type seizure with tonic-clonic phases and unconsciousness lasting several minutes. It does not induce a (benign) faint, and use of the system might cause structural brain damage. Thus, this system is unlikely to be acceptable under Jewish religious law and its animal welfare value can be questioned.

## Introduction

Throughout history, a principal method of slaughtering animals for food has been some form of exsanguination. In recent centuries the perception developed that such a method when used alone results in suffering for the animal that could be alleviated to some degree by means of a prior action, now known as ‘pre-cut stunning’, that renders the animal unconscious. Pre-cut stunning is now compulsory in most Western countries, including in the European Union (EU). The basic methods of stunning employed today are as follows: electrical stunning, penetrating captive bolt, non-penetrating captive bolt, and gas. All of these methods have been deemed unacceptable according to normative Jewish law for use prior to kosher slaughter (*shechita*) (for a discussion, see Zivotofsky 2012). In response to several jurisdictions banning all non-stun slaughter and other regions severely restricting it, there is pressure to develop a stunning system that would be acceptable to Jewish religious authorities. In recent years a new system has been designed, and its inventors are advocating its use for *shechita* and Halal. It is in response to that suggestion that this review of the evidence was carried out and this opinion written.

The evaluation of the system presented herein is primarily based on a recent peer-reviewed paper (Small *et al.* 2019) and the developer’s Final Report (prepared by Small 2021). While preliminary data on the system were previously reported (Small *et al.* 2013, 2015; Rault *et al.* 2014; McLean *et al.* 2017), Small *et al.* (2019) is a comprehensive report in that it reviews the previous findings and reports on additional behavioural and EEG results of two trial runs of the system. The animal experiments are detailed and thoroughly described in the paper and report. The engineering behind the system, including the custom-made waveguide, have undergone extensive development and modification and is sophisticated.

## Description of the system

In recent years an alternative to current methods of stunning cattle has been introduced to the animal welfare community and presented to rabbinic authorities and the kosher slaughter industry in Israel, Europe, the US, and Australia for evaluation as a means to stun cattle before *shechita*, with the assertion that this method should be acceptable according to Jewish religious law (*halacha*) and Muslim Sharia law. The system, named DTS: Diathermic Syncope®, was developed by two Australian companies (Wagstaff Food Service Pty Ltd and Advanced Microwave Technologies), and has been in development since at least 2009. The developers have stated in page 4 of their Final Report (Small 2021) that: “*The project was planned and executed under end-user-centred design principles, with the Halal and Kosher markets in mind*”, thus a goal of the developers was to produce a system acceptable to the kosher and halal consumer.

In introducing the system, the developers point out flaws in the currently used stunning systems, problems which also explain why the Jewish community has been hesitant to accept those methods. They then explain that two additional potential methods of attaining loss of consciousness (LOC) are general anaesthetics and fainting. They rule out the use of general anaesthetics in slaughter of animals for food because of the residual pharmaceuticals in the tissues and that its use would significantly slow the line speed. They therefore aimed to develop a system that would cause the animal to faint, or as it is technically called, ‘induce syncope’, and the resulting system was trademarked as Diathermic Syncope®. Diathermy is, as defined in the Harvard on-line medical dictionary (https://www.merriam-webster.com/dictionary/diathermy#medicalDictionary): “*Use of high-frequency electric currents to heat deep muscle and joint tissue as a form of physical therapy*” and the Merriam-Webster Medical dictionary (https://www.merriam-webster.com/dictionary/syncope): “*the generation of heat in tissue by electric currents for medical or surgical purposes.*” In other words, diathermy is, among other things, the heating up of body tissue using electromagnetic waves.

Syncope is fainting or, as defined by Merriam-Webster (https://www.merriam-webster.com/dictionary/syncope): “*loss of consciousness resulting from insufficient blood flow to the brain: faint*”, and in the Harvard online dictionary (https://www.health.harvard.edu/q-through-z#S-terms) as “*syncope: Fainting or loss of consciousness caused by a temporary shortage of oxygen in the brain.*”

Before reviewing the animal welfare and associated Jewish legal aspects, it is important to determine what the system is and is not doing, and if what is really happening to the cows is in fact fainting brought on by diathermy.

In basic terms, the system directs electromagnetic energy at a frequency of 922 MHz at energies of about 200 kJ using a waveguide towards the cow’s brain. The purpose of this is to raise the temperature of the brain to the point where it ceases normal functioning, and the animal loses consciousness.

A basic description of how this system works could be that it is like putting the cow’s brain in a microwave oven to heat it up until the brain stops working properly. Indeed, consumer microwave ovens use waves at about 2.45 GHz and large industrial/commercial microwave ovens often use waves at approximately the same frequency as this system, 915 MHz. Microwaves warm food in a microwave oven and, in this system, the brain of the cow by causing the water molecules in the tissue to vibrate, thereby producing heat. Food heats more than a bowl, because it has a significantly higher water content than the bowl. Similarly, the brain warms because it has a relatively high water content: white matter is approximately 69% water and grey matter 84% (Oros-Peusquens *et al.* 2019). Mitchell *et al.* (1945) estimated average water content of the brain to be about 73%, which is more than skin (about 64%) and far greater than bone (31%). Aiming the microwaves at the brain will cause it to heat even if some of the waves are blocked by the skull and skin (McLean *et al.* 2017).

In the introduction to Small *et al.* (2019) it states: “*The aim of the system is to selectively increase the temperature of the brain, to the point that hyperthermic syncope [fainting] occurs.*” But does this heating of the brain actually result in syncope? Syncope has a specific medical/scientific definition as per, for example, The National Institute of Neurological Disorders and Stroke (NINDS) on their website (https://www.ninds.nih.gov/health-information/disorders/syncope): “*a medical term used to describe a temporary loss of consciousness due to the sudden decline of blood flow to the brain. Syncope is commonly called fainting or ‘passing out.’*” This definition is expanded on in the official definition of the Task Force on Syncope of the European Society of Cardiology (ESC): “*a transient, self-limited loss of consciousness, usually leading to falling. The onset of syncope is relatively rapid, and the subsequent recovery is spontaneous, complete, and relatively prompt. The underlying mechanism is a transient global cerebral hypoperfusion*” (Brignole *et al.* 2001). The DTS system does not appear to cause cerebral hypoperfusion, which is a drop in blood flow to the brain, and therefore this is not what is leading to the loss of consciousness. *Shechita*, the Jewish religious method of animal slaughter, does initiate a precipitous drop in blood pressure and blood perfusion of the brain, bringing about a (permanent) loss of consciousness (Rosen 2004).

Transient LOC can be divided into traumatic and non-traumatic; and within non-traumatic, syncope is only one of many potential causes (see van Dijk *et al.* 2020). Even if LOC caused by DTS is transient, that does not make it fit a definition of syncope. Van Dijk *et al.* (2009) explain that: “*transient loss of consciousness [TLOC] is defined as an apparent loss of consciousness with an abrupt onset, a short duration, and a spontaneous and complete recovery. Syncope is defined as TLOC due to cerebral hypoperfusion and is divided into reflex syncope [synonymous with neurally mediated syncope], syncope due to orthostatic hypotension, and cardiac syncope [arrhythmic or associated with structural cardiac disease].*” That same year, Wieling *et al.* (2009) succinctly describe syncope in a similar fashion: “*A failure of the systemic circulation to perfuse the brain sufficiently results in a stereotyped progression of neurological symptoms and signs culminating in loss of consciousness; when transient, this is syncope.*” They describe various types of spontaneous syncope and then detail eight ways to induce syncope; heating the brain is not one of them.

In the Final Report (Small 2021) from the DTS developers (p 9), the authors again term what they induce as “hyperthermic syncope”, claiming that “*The mechanism of action is selectively increasing the temperature in the brain to the point that hyperthermic syncope [fainting] occurs.*” So what is hyperthermic syncope? Classic hyperthermic syncope or “heat syncope” is fainting that results from an overheating of the whole body, leading to a variety of responses, including the dilation of arterioles in the skin in order to radiate heat, resulting in diminished blood flow to the brain and classic fainting. The temperature of the brain itself usually does not increase much above normal operating temperatures, as the dilating of the blood vessels occurs specifically in order to protect the most vital organ, the brain, by ensuring that it does not overheat. This compensatory mechanism is actually quite dramatic; under heat stress conditions human skin blood flow is estimated to increase from ∼300 ml min^–1^ upward to 7.5 L min^–1^, resulting in the capacity for 50% or more of cardiac output being directed to the skin (Crandall *et al.* 2010). That alone does not account for the hyperthermic syncope, as Nelson *et al.* (2011) explain: “*While the mechanism[s] remains unclear, it is well established that cerebral blood flow [CBF] is reduced during passive heat stress. Indeed, any condition that jeopardises CBF will ultimately lead to syncope.*” Thus, hyperthermic syncope, like all syncope, is caused by reduced blood flow to the brain. The mechanism in DTS wherein the brain becomes heated, and the loss of consciousness occurs as a result of brain dysfunction is fundamentally different from classic ‘hyperthermic syncope.’

Another indication that DTS does not cause fainting is the time to regaining consciousness. There are many causes of syncope with a range of duration of LOC, but in general, recovery from fainting typically occurs within seconds, and certainly less than a minute, as observed by van Dijk *et al.* (2009): “*Epileptic seizures usually last 1 min or more, and are usually longer than syncopal attacks*” and, again in van Dijk *et al.* (2020): “*Syncope usually lasts less than a minute…‥.*” The claim by the developers of the DTS system is that it is superior to electrical stunning specifically because DTS produces a long-duration unconsciousness. They note that in electrical stunning the duration of unconsciousness is 31–90 s while in DTS it is between 100 and 240 s. Later in the report they give between 2 and 4.5 min as the time to start of recovery.

It should be noted that much of the cited data are based on human studies. Fainting is of greater concern in humans than other animals and thus more studies on fainting are carried out on humans. In addition, humans tend to faint far more frequently than other animals and this has been blamed on two evolutionary novelties: the proportion of cardiac output going upwards to the brain is much greater in humans, and humans’ relatively large legs suggest that the volume lost to venous pooling is also larger (van Dijk 2003). Nonetheless, it is generally assumed that the characteristics of fainting are similar across species.

Given the above, it would appear that DTS, despite its name, does not in fact induce hyperthermic syncope, nor syncope (fainting) at all. Both hyperthermic syncope and DTS involve loss of consciousness because of heat and because of brain malfunction, but that does not mean that DTS causes hyperthermic syncope. This is not merely an issue of semantics – as the term syncope evokes an image of the painless and reversible phenomenon that is fainting.

## Is the DTS system good from an animal welfare perspective?

Based on the above it seems that DTS does not cause simple fainting, but it may still be an approach to reduce welfare compromise during the slaughter process, and thus might be a system worth considering. In discussing the elevated temperature of the cow’s forehead after DTS use, the Final Report (pp 15, 25) (Small 2021) observes that “*Australian cattle may experience ambient air temperatures of 45° Celsius in summer and up to 50° Celsius in direct sunlight.*” This again highlights the distinction between hyperthermic syncope and DTS. DTS selectively heats the brain and *inter alia*, the skin, which may then reach temperatures of 45–50°C. In the hot Australian summer, on the other hand, the heating is taking place due to an increase in the ambient temperature. In that situation, because the brain is such a significant organ, the body has compensatory thermoregulatory mechanisms to maintain stability of internal temperatures preventing the brain from overheating. The brain temperature is very resistant to external temperature and brain temperature is usually minimally affected by environmental temperatures. Thus, the temperature of the brain of a cow in a hot Australian summer, even in the direct sun, should not increase very much, while an increase of several degrees at the skull after application of DTS indicates a significant increase in brain temperature. Of course, the cows know to seek shade, as Edwards-Callaway *et al.* (2021) report: “*Cattle will seek shade when available; when the shade is not available, cattle orient their body position in a way that reduces surface area exposure to solar radiation.”* And, indeed, most cows will respond with various means to adapt to heat stress, methods that are usually successful. If these and other adaptive responses are not effective, the animal’s body temperature and eventually brain temperature will rise, which can lead to heatstroke and death, a phenomenon very different from hyperthermic syncope and more like what DTS does.

In Small *et al.* (2019), in Trial 2 (the more complete trial, with an improved system), 20 animals received the DTS treatment. Of these, three did not lose consciousness and of the 17 that did, four died from it. In other words, according to the company’s own data, at best 13 out of 20 animals were rendered unconscious in a manner that might be reversible. They explain the three failures as relating to difficulties in maintaining contact between the waveguide and the cow’s head, a problem they explain as relating to the “extreme contouring of the forehead.” This is similar to the sort of problem they themselves raised (p 7) regarding the current stunning systems, such as the captive bolt, of which they observe, “*it can be difficult for the operator to accurately position the ‘shot.’*” Thus, with both systems, variability in the individual bovine anatomy presents a challenge in achieving a successful stun. This was also mentioned in Small *et al.* (2019) where they note that, for example, forehead skin thickness in the bulls studied ranged from 5 to 24 mm, a significant variation that presumably would affect the initial setting of input parameters for their system, as the microwaves need to penetrate the skin in order to reach the brain.

As described in the Final Report (Small 2021), there seems to be a narrow window of energy and power settings which result in successful DTS stunning. Outside that window, the settings are either too low, which may cause suffering to the cow similar to that experienced by people experiencing heat stroke, or too high, which can result in skin burns, intense convulsions, and even death. Regarding the former scenario, the Final Report (Small 2021) reported on 258 animals of which 24 were not successfully rendered unconscious because of “*equipment failure resulting in generator shutdown and the required energy not being applied*” or low energy levels, describing a situation in which nearly 10% of the cows had their brains microwaved insufficiently to render them unconscious but possibly sufficiently to cause suffering. Depending on the characteristics of the individual animal, it is difficult to ascertain the likelihood of fitting into the successful stunning window, but the data from Small *et al.* (2019) and the Final Report (Small 2021) indicate that the DTS system currently is ineffective or overpowered in 9–35% of attempts. This sounds similar to one of the issues regarding electrical stunning – the inability to consistently get the ‘right’ conditions (Zivotofsky & Strous 2012).

Using the data (Table 2) for the 20 cows in Trial 2 in Small *et al.* (2019) when the improved system was used and taking time to collapse as a measure of LOC, the median time to LOC can be calculated as 5 s (range: < 1–19) and the mean (± SD) as 6.25 (± 4.4) s, although for some animals it took much longer. During the period leading up to LOC, the authors describe back arching and neck muscles contracting in the animals. From human experience, when a person begins to experience heatstroke and the brain itself begins to heat up, they feel unwell with muscle cramps, spasms, and pain as well as severe headaches. Thus, while the mechanism and time-frame are different and thus this comparison is merely suggestive, it is possible that the animal, although the rest of the body is at near-normal temperature, will suffer intense headaches and pain as its brain is heated. This possibility exists because, unlike the goal in successful captive-bolt or electric stunning in which LOC is instantaneous (or near instantaneous), DTS as a method is not instantaneous and thus there is the potential for pain and distress prior to LOC. This is starkly highlighted by the same Table 2 in the first trial in which there were seven cows on which captive bolt was used and for all of them time to collapse is given as < 1 s.

It is important to ask what is actually happening to the cow when the DTS system is used. From a clinical and electrophysiological perspective, its end result appears to be the same as electrical stunning – causing a large seizure, as acknowledged in the Final Report (Small 2021), albeit only in the detailed section and not in the broader statements. It states (section 3.1.2.2; p 14): “*After application, these animals demonstrated behavioural and EEG signs consistent with an electrical stun.*” In section 3.3 of Small *et al.* (2019) it states: “*In Trial 2, using the improved restraint and energy delivery apparatus, the 17 animals that were assessed as insensible following DTS application, demonstrated behavioural signs consistent with an electrical stun – rapid blinking or flicking of the eyelids including the membrane nictitans [third eyelid], abrupt loss of posture, loss of rhythmic breathing, tonic [stiff] and clonic [convulsive] phases. As the behavioural signs obtained using DTS, particularly in Trial 2, appear to be similar to those produced by electrical stunning…*” In the Final Report (Small 2021; p 24): “*The stunned animals demonstrated behavioural signs consistent with an electrical stun*” and in Table 3 they list “*Epileptiform seizure*” as occurring in DTS and electrical stunning. In section 3.4 of Small *et al.* (2019) they say that the EEG showed “*seizure-like activity in all DTS-treated animals with the exception of animals [three animals listed].*” This was already recognised early in the development, as it states in Rault *et al.* (2014): “*Microwave energy can induce insensibility in cattle based on seizure-like complexes in the EEG.*” When a physician expert in seizures (RG, personal communication 2022) looked at the EEG traces in the Final Report Small (2021), their immediate reaction was that the system seemed to induce a seizure very reminiscent of electrical stunning. The EEGs were included by the authors in order to demonstrate to the reader that the animal has lost consciousness. However they also show that the animal has experienced a seizure. DTS causes LOC – not by inducing fainting – but rather by altering brain function in a manner similar to electrical stunning and resulting in a seizure reminiscent of that caused by electrical stunning.

From an animal welfare perspective, the DTS system would seem to have some of the flaws of traditional stunning techniques, including, as evident from the data presented, that there are inevitably going to be a significant number of ‘mis-stuns’ in which the animal may suffer. Whether the animal also suffers more than in other slaughter methods when there is a successful application is unknown. But it is certainly not claimed to be instantaneous LOC as is the goal with electrical and captive-bolt stunning. Nor is the description of the animal’s behaviour in the period before LOC one of calmness. For example, as noted in Small *et al.* (2019) “*some animals [particularly animals 1.10, 1.14 and 1.16] the back arched and the muscles of the neck contracted, pulling the chin down into the chin lift. This occurred at about 2–3 s following the start of treatment.*” In short, the DTS system does not induce fainting nor appear to guarantee an approach to slaughter that reduces to a minimum the welfare compromise that occurs during the slaughter process.

## Is DTS acceptable from a Jewish religious perspective?

According to Jewish law, for a cow to be considered kosher, it must not have any physical defects and must be slaughtered whilst still alive (for details, see Zivotofsky 2010). It is because of concerns for these two issues that pre-cut stunning is problematic for *shechita.*

The significant increase in brain temperature caused by the DTS system could lead to permanent, physical damage that might render the animal non-kosher. In his comprehensive review of the subject of brain-heating, Kiyatkin (2019) notes: “*…temperature increases or decreases exceeding physiological range (35–39°C) can adversely affect brain cells and neural functions. While brain cells seem to tolerate low temperatures well, multiple* in vitro *studies suggest that high temperature (> 40.0°C) has destructive effects on various types of brain cells”* he further notes that *“pathological brain hyperthermia that significantly alters neural functions and may induce structural damage to brain cells.”* Utilising drug studies, he further states that “*Robust hyperthermia is a known life-threatening complication of overdose of psychomotor stimulants such as MDMA and methamphetamine [METH]. I will demonstrate that the brain temperature effects of these drugs are strongly modulated by high activity states and adverse environmental conditions that limit heat dissipation, thus leading to pathological brain hyperthermia that induce multiple functional and structural brain changes that could lead to lethality.*”

In a 2015 conference presentation, (https://www.icnirp.org/cms/upload/presentations/Thermo/ICNIRPWHOThermo_2015_Kiyatkin.pdf), Kiyatkin (2015) observed that “*Brain tissue is exceptionally sensitive to heat – structural changes occurring with a 3–4°C increase above normal baseline.*” The damage greatly progresses with slight temperature increases. The DTS system causes not a slight but a significant increase in the brain temperature. The changes in brain temperature above its physiological range (>39°C) could induce functional and, also important from the kosher perspective, morphological brain abnormalities. The brain hyperthermia leads to an increase in brain volume causing damage to brain capillaries. These changes will not be easy to detect, but based on previous research, almost certainly take place (Kiyatkin 2015, 2019, and private communications from EA Kiyatkin 2021).

Guy and Chou (1982) exposed 70 rats to 915 MHz pulsed magnetic fields that raised the brain temperature by a maximum of 8°C to a maximum of about 46°C and led to transient LOC of 4- to 5-min duration. Even this short duration of the brain being significantly above its physiologic temperature was sufficient for histological examination of a small subset of those rats, one day after exposure, to reveal microscopic changes of unilateral focal and microfocal encephalomalacia due to nerve demyelination; a month after exposure, a macroscopic change of brain swelling was noted along with microfocal glial nodules. In one of the early DTS papers (McLean *et al.* 2017) it was explained that a minimum increase of 6°C was required to induce stunning, and the authors were pleased to be able to achieve temperature increases of 8°C. Thus, there is a realistic concern that the DTS system renders the animal non-kosher due to physical damage of the brain.

Certain jerky movements of the limbs following slaughter of “sickly animals” are described in the religious codes as a sign the animal was alive when slaughtered, and absence of these movements renders the meat non-kosher. In the description of the DTS system, the animal will not exhibit the jerky leg movements following the *shechita* because, as is described in the Final Report (Small 2021; p 17):

“*Cattle that are stunned using DTS [or for that matter, electrical stunning] can quickly enter a stiff, tonic ‘rocking-horse’ position, with all four legs rigidly extended… After this ‘rocking horse’ phase [many tens or hundreds of seconds later], the stunned animal develops a convulsive or kicking phase [similar to that seen in an epileptic episode], followed by a recovery phase. In commercial processing it is important to exsanguinate the animal before the kicking phase begins – both to ensure that the animal does not recover during bleed-out, and because the size of the animal makes handling during that kicking phase very dangerous for the operator.*”

It is worth noting that the authors recognise that a tonic-clonic phase response is far more similar to an induced epileptic-type seizure than to a syncope.

Despite the attempts of the DTS system designers to distance themselves from current stunning systems, slaughter carried out following DTS use far more closely resembles slaughter done with some of the other stunning systems than it does non-stun slaughter. In fact, as demonstrated above, they even note several times in their report that DTS produces results similar to electrical stunning, a system long rejected by Jewish religious authorities.

## Summary and Conclusion

The DTS: Diathermic Syncope® System is a relatively new and innovative technique for the stunning of cattle prior to slaughter. It focuses radiation in the form of microwave energy directly towards the cow’s brain, thereby raising the brain temperature, leading to the animal being rendered unconscious. Unlike what the name implies, the animal does not faint; rather it appears to experience an epileptic-type seizure and loses consciousness due to functional and morphological changes brought about by the brain hyperthermia. This is not mere semantics but a very important issue. Fainting is viewed by the general public as a benign way to lose consciousness because it is seen as being painless and devoid of suffering. Thus, if DTS really induces syncope (fainting) it would be viewed as animal welfare-friendly; if it were causing LOC by another means, e.g. heating the brain until the animal seizes, the system might lose a lot of its general appeal. And it is not only public perception. Since DTS is not designed to induce instantaneous LOC but rather to gradually heat the brain until LOC and seizures occur, there is also the potential for compromising animal welfare.

As the brain is such a sensitive organ, it seems likely, based on the data presented above, that brain damage, certainly at the cellular level, takes place due to this brain hyperthermia. Whether this damage would be noticeable on gross anatomical observation is uncertain, but there certainly is at least a potential concern that the animal has been made unsuitable for kosher consumption, because excessive heating of the brain (as occurs with DTS) can cause brain swelling and thereby perforation of the meninges as well as liquefactive necrosis leading to observable physical damage.

In summary, the use of this new system, the DTS: Diathermic Syncope® system, raises animal welfare and religious concerns and should not be accepted for use by either secular authorities or rabbis based on it causing a benign faint – because it does not. It leads to unconsciousness via a mechanism similar to electrical stunning, and thus will likely not be accepted for use prior to kosher slaughter and requires further evaluation by the animal welfare community before being accepted as a humane stunning system.
